# *Caldanaerobacter subterraneus* subsp. *keratinolyticus* subsp. nov., a Novel Feather-Degrading Anaerobic Thermophile

**DOI:** 10.3390/microorganisms12071277

**Published:** 2024-06-23

**Authors:** Akzhigit Mashzhan, Aida Kistaubayeva, Rubén Javier-López, Akerke Bissenbay, Nils-Kåre Birkeland

**Affiliations:** 1 Department of Biotechnology, Al-Farabi Kazakh National University, Al-Farabi Av. 71, 050040 Almaty, Kazakhstan; kistaubayeva.kaznu@gmail.com (A.K.); akerke.bissenbay@gmail.com (A.B.); 2 Department of Biological Sciences, University of Bergen, P.O. Box 7803, NO-5020 Bergen, Norway; ruben.javier-lopez@uib.no; 3 Science Research Institute of Biology and Biotechnology Peoblem, Al-Farabi Kazakh National University, Al-Farabi Av. 71, 050040 Almaty, Kazakhstan; 4 Almaty Branch of National Center for Biotechnology in Central Reference Laboratory (CRL), Zhahanger St. 14, 050054 Almaty, Kazakhstan; 5 Department of Botany, E.A. Buketov Karaganda State University, Universitet St. 28, 100028 Karaganda, Kazakhstan

**Keywords:** anaerobe, geothermal spring in Kazakhstan, feather degrading, thermophile

## Abstract

*Caldanaerobacter subterraneus* subsp. *keratinolyticus* subsp. nov. strain KAk was isolated from a geothermal hot spring located in Kazakhstan. Growth occurred at temperatures ranging from 50 to 80 °C, with approximately 70 °C as optimum. It also thrived in pH conditions ranging from 4.0 to 9.0, with the best growth occurring at 6.8. Under optimal conditions in a glucose-containing medium, the cells were predominantly observed singly, in pairs, or less frequently in chains, and did not form endospores. However, under conditions involving growth with merino wool or feathers, or under suboptimal conditions, the cells of strain KAk exhibited a notably elongated and thinner morphology, with lengths ranging from 5 to 8 µm, and spores were observed. The KAk strain exhibited efficient degradation of feather keratin and merino wool at temperatures ranging from 65 to 70 °C. Analysis of the 16S rRNA gene sequence placed KAk within the genus *Caldanaerobacter*, family *Thermoanaerobacteraceae*, with the highest similarity to *C. subterraneus* subsp. *tengcongensis* MB4^T^ (98.84% sequence identity). Furthermore, our analysis of the draft genome sequence indicated a genome size of 2.4 Mbp, accompanied by a G+C value of 37.6 mol%. This study elucidated the physiological and genomic characteristics of strain KAk, highlighting its keratinolytic capabilities and distinctiveness compared to other members of the genus *Caldanaerobacter.*

## 1. Introduction

The genus *Caldanaerobacter* is among 18 validly published genera in the family *Thermoanaerobacteraceae* [1]. To date, it comprises two species: *Caldanaerobacter uzonensis* K67^T^ [2] and *C. subterraneus*, including *C. subterraneus* DSM 13054^T^ [3,4], *C*. *subterraneus* subsp. *pacificus* JM^T^ [5], *C. subterraneus* subsp. *tengcongensis* MB4^T^ [6], and *C*. *subterraneus* subsp. *younseiensis* KB-1^T^ [7,8]. *Caldanaerobacter* spp. are strictly anaerobic thermophiles that may exhibit either a positive or negative Gram reaction, and endospores can be observed [4]. These bacteria possess a fermentative metabolism and have been identified in diverse geothermal habitats, including hot vents, geothermal hot streams, oilfields, and terrestrial hot springs [2,4]. Members of the genus *Caldanaerobacter* utilize a range of complex substrates such as xylan, starch, and sugar-containing compounds. However, to date, no members of this genus have been described to have the capacity to degrade keratin.

Keratins are insoluble, sulfur-containing fibrous proteins mostly found in the epithelial cells of vertebrates. They are the primary components of outer structural features such as hair, feathers, horns, nails, and wool. Owing to these structural characteristics, the high content of intramolecular cysteine disulfide and hydrogen bonds makes keratin extremely resistant to biological and chemical degradation [9]. Keratin is categorized into alpha and beta forms [10]. Alpha keratins are found in the epidermis of most mammals (e.g., wool and hair), are rich in alpha helical secondary structures, and have subunit molecular masses of 40–70 kDa [11]. The size of beta keratins found in the skin of birds and reptiles is normally smaller than that of alpha keratins (10–20 kDa) [11]. Approximately 10 million tons of keratin waste are generated annually worldwide, with feather waste constituting approximately 8.5 million tons. As feathers contain a wealth of essential amino acids (70%), vitamins, growth factors, and some high-value elements, they are considered a quality protein feed source with unique value [12]. Feathers can be hydrolyzed by mechanical or chemical treatments to obtain fertilizers, feedstock, or glues. Nevertheless, keratin is only incompletely degraded by these methods, which are expensive and can cause harmful environmental contamination [13,14]. At present, most keratin-containing biowastes are disposed by burying in landfills, burning, or treating at high temperatures, where they turn into low-quality animal flour, and are used as a protein supplement in feed mixtures of domestic animals and fish. However, it has been established that this flour can serve as a carrier of prions, responsible for certain related disease such as mad cow and Creutzfeldt-Jacob diseases and bovine spongiform encephalopathy [10]. The decomposition of feather biomass through chemical or hydrothermal processes results in the decomposition of essential amino acids such as serine, methionine, lysine, cysteine, and proline [15]. Therefore, the biodegradation of keratin-containing biowaste using keratin-degrading microorganisms is a more effective, suitable, and inexpensive approach for waste management and environmental and human safety [16]. Moreover, the transformation of low-priced and easily obtainable chicken feathers into dietary proteins for animal feedstock supports a bioeconomic approach [17,18]. Therefore, the present study aims to characterize a novel subspecies of *Caldanaerobacter subterraneus* isolated from a hot spring in Kazakhstan. This subspecies demonstrates effective degradation of native chicken feathers under anaerobic conditions at temperatures reaching up to 70 °C. This is the first report of a keratinolytic member of this genus.

## 2. Materials and Methods

### 2.1. Sampling and Cultivation

The sample was obtained from the Zharkent geothermal spring, Almaty region, Kazakhstan (43°58.79″ N, 79°40.79″ E), with a temperature of 76 °C, pH 6.8, and a depth of 0.8 m [19]. Mixed samples of water and sediment were placed in 100 mL anaerobic serum flasks, tightly sealed, and then transported to the laboratory at 4 °C.

A modified Hungate technique was used to prepare anaerobic enrichment cultures [20]. Enrichment and cultivation were performed using the freshwater mineral medium (MMF) containing the following ingredients (per liter): NaCl, 1.0 g; KCl, 0.3 g; MgSO_4_·7H_2_O, 0.3 g; NH_4_Cl, 0.5 g; KH_2_PO_4_, 0.3 g; and CaCl_2_·2H_2_O, 0.1 g. A trace element solution (1 mL) was also added, containing (per liter): FeCl_2_·4H_2_O, 1.5 g; HCl (25%), 10 mL; CoCl_2_·6H_2_O, 190 mg; MnCl_2_·H_2_O, 100 mg; Na_2_MoO_4_·2H_2_O, 36 mg; ZnCl_2_, 70 mg; NiCl_2_·6H_2_O, 24 mg; CuCl_2_·H_2_O, 2 mg; L-Cysteine-HCl·H_2_O, 0.25 g; and H_3_BO_3_, 6 mg. At the end, 0.5 mL of 0.2% resazurin was included in the medium as a redox indicator. Following autoclaving at 121 °C for 20 min and cooling to around 50 °C under continuous nitrogen gas flushing, 10 mL of vitamin solution containing the following ingredients was added: D (+) biotin, 2 mg/L; 4-aminobenzoic acid, 8 mg/L; nicotinic acid, 20 mg/L; pyridoxamine 2HCl, 30 mg/L; Ca-D (+) pantothenic acid, 10 mg/L; thiamine dichloride, 20 mg/L; and vitamin B12, 10 mg/L. Subsequently, the pH was adjusted to 6.5 using 2 M HCl. The medium was then transferred aseptically to 100 mL serum flasks using the Hungate technique and sealed with butyl rubber stoppers and metal crimps [20]. Finally, 0.1% yeast extract was supplemented into the medium.

Enrichment was conducted in 50 mL serum flasks containing 30 mL MMF supplemented with yeast extract. The media were inoculated with 1 mL water and sediment samples and incubated at 70 °C without agitation for four days.

The cultures were purified using the dilution-to-extinction technique, resulting in the isolation of the bacterial strain designated KAk.

Eight flasks were prepared, each containing 9 mL of the MMF supplemented with yeast extract. Starting with the original culture, 1 mL was transferred to one of these flasks to create a 10^−1^ dilution. Subsequently, ten-fold dilutions were made successively from this flask, culminating in a series of dilutions of up to 10^−9^. All the flasks were incubated at 70 °C for 48 h. This process was repeated with the inoculum from the highest dilution that exhibited growth. The culture demonstrating growth at the highest dilution was examined under phase-contrast microscopy and appeared to be pure. This culture served as the stock culture for all subsequent experiments.

### 2.2. Microscopy

The morphological features of the isolate KAk, cultivated on MMF at 70 °C for 24 h, were analyzed using both a phase-contrast microscope (Eclipse E400; Nikon, Minato City, Japan) with an oil immersion lens and a Nikon DS-Fi3 camera. Additionally, a scanning electron microscope (Jeol JSM-7400F; JEOL, Tokyo, Japan) at the Molecular Imaging Center (MIC) of the University of Bergen (https://www.uib.no/en/rg/mic; accessed on 24 August 2023) was employed for further examination. Samples were prepared as per a previously described protocol [21].

### 2.3. Physiological Characterization

The temperature, pH, and NaCl ranges for growth were determined by incubating the isolate in MMF. The medium in the serum flasks was adjusted to the desired pH, and the pH was measured at ambient temperature by injecting diluted sterile anaerobic stock solutions of NaHCO_3_ (1 M) or Na_2_CO_3_ (1 M). The growth temperature range was assessed at intervals of 5 °C from 30 to 90 °C. NaCl was weighed directly in a flask before being dispensed into the culture medium. The strain was subcultured at least once under the same experimental conditions prior to the determination of growth rates. API 20 A and API ZYM tests (SKU:20300 and SKU:25200, respectively; BioMérieux, Inc., Marcy-l’Etoile, France) were conducted following the manufacturer’s instructions. The hydrolysis of various specific substrates, including starch, sucrose, galactose, xylan, pyruvate, arabinose, xylose, glucose, peptone, CM-cellulose, and merino wool, was determined under strict anoxic conditions using MMF. After 24 and 48 h of incubation at 70 °C, growth was assessed using phase-contrast microscopy. Growth was considered if cell density doubled within 24 h, whereas slow growth was indicated by the doubling of cell density within 48 h. All experiments were conducted in triplicate with corresponding negative and positive controls.

### 2.4. 16S rRNA Gene Sequence Analysis

The bacteria cultured in MMF for 24 h were collected by centrifugation at 4 °C for 15 min at 6000× *g* (8000) rpm. Genomic DNA extraction and purification were performed using the GenElute Bacterial Genomic DNA Extraction Kit (NA2100; Sigma-Aldrich, St. Louis, MO, USA) and GenElute PCR Cleanup Kit (NA1020; Sigma-Aldrich). The 16S rRNA gene was amplified using PCR with the universal bacterial primers 27F and 1525R, as described previously [21]. The PCR products were sequenced using Sanger sequencing (Big-Dye 3.1 Terminator kit (Perkin Elmer, Waltham, MA, USA) on an ABI PRISM capillary sequencer at the University of Bergen Sequencing Facility (https://www.uib.no/en/seqlab, accessed on 21 May 2023). The sequence reads underwent validation and correction using the MEGA v11 software suite [22]. Subsequently, they were merged using the EMBOSS software v6.6.0.0 package [23]. The obtained 16S rRNA gene sequences were compared to entries in public databases using BLASTN with default parameters (https://blast.ncbi.nlm.nih.gov/Blast.cgi, accessed on 5 May 2023). Multiple sequence alignments were performed using CLUSTAL X [24]. Phylogenetic analyses were conducted using the maximum likelihood method in the MEGA v11 software suite [22]. Evolutionary distances were computed using the Tamura-Nei model [25]. Bootstrap analysis of the maximum likelihood data involved 1000 resampling iterations to evaluate confidence in the branching points [26]. In addition, positions with missing data were excluded from the analysis. The 16S rRNA gene sequence of *Caldicellulosiruptor saccharolyticus* served as an outgroup.

### 2.5. Genome Sequencing and Phylogenomics

The genome of the KAk strain was sequenced using shotgun Illumina sequencing by a commercial next-generation DNA sequencer (Eurofins Genomics, Ebersberg, Germany) employing paired-end technology. Quality control and trimming of raw reads were conducted using the FastQC v0.12.1 [27] and Trimmomatic v0.36 tools [28], respectively, to ensure the data’s integrity and reliability. The trimmed sequences were assembled using SPAdes v3.15.3 [29] de novo assembly tool and subsequently uploaded to the RAST 2.0 [30,31] and NCBI servers for analysis and annotation. Completeness was checked using CheckM v1.0.18 [32]. Whole-genome shotgun projects have been submitted to GenBank and assigned the accession number JAULJF000000000. Genome-based phylogenetic analysis was performed using the Type (Strain) Genome Server (TYGS) available on the DSMZ website (http://ggdc.dsmz.de/, accessed on 23 August 2023) [33]. Additionally, the Ortho Average Nucleotide Identity (OrthoANI) between the KAk strain and type strains of *C. subterraneus* was performed with OAT (OrthoANI Tool) [34]. The Blast Ring Image Generator (BRIG) was utilized to compare the genome of the KAk strain to that of the closest type strain [35].

### 2.6. Feather Degradation Test 

Native chicken breast feathers were used as substrates for keratin degradation tests. The feathers were thoroughly rinsed multiple times with deionized water to eliminate dust and skin particles. Subsequently, they were dried in an oven for 24 h at 50 °C. After drying, 25 ± 5 mg of the chicken feathers was added to 25 mL of anaerobic medium containing the following ingredients per liter: NaCl, 1.0 g; yeast extract, 0.01 g; L-Cysteine-HCl·H_2_O, 0.25 g; and resazurin (0.2%), 0.5 mL. Additionally, 1 mL of the KAk strain inoculum was added to the medium. A flask containing only medium with feathers without any inoculation served as the negative control.

The cultures were incubated anaerobically at 70 °C. Every 24 h, the flasks were visually examined to monitor changes in the integrity of the feather.

The biodegradation of feathers by KAk during fermentation was assessed by measuring the percentage of substrate weight loss. After fermentation, the remaining feather residue was separated and collected from the culture supernatant via filtration. The collected residual feathers were thoroughly washed with deionized water 2–3 times to eliminate soluble substances and attached bacteria. Finally, the washed feathers were dried in a hot air oven at 50 °C for 48 h. The extent of feather biodegradation was quantified as the percentage of weight loss relative to the initial dry weight of the substrate (before and after incubation). A control flask containing uninoculated feathers was used as a reference for the assessment. The feather degradation rate was calculated using the following formula: feather degradation rate (%) = 100 × (B − A)/B, where B is the initial weight of the feathers before degradation and A is the final weight of the feathers after degradation [12].

## 3. Results

### 3.1. Isolation and Morphology

**Figure 1:**
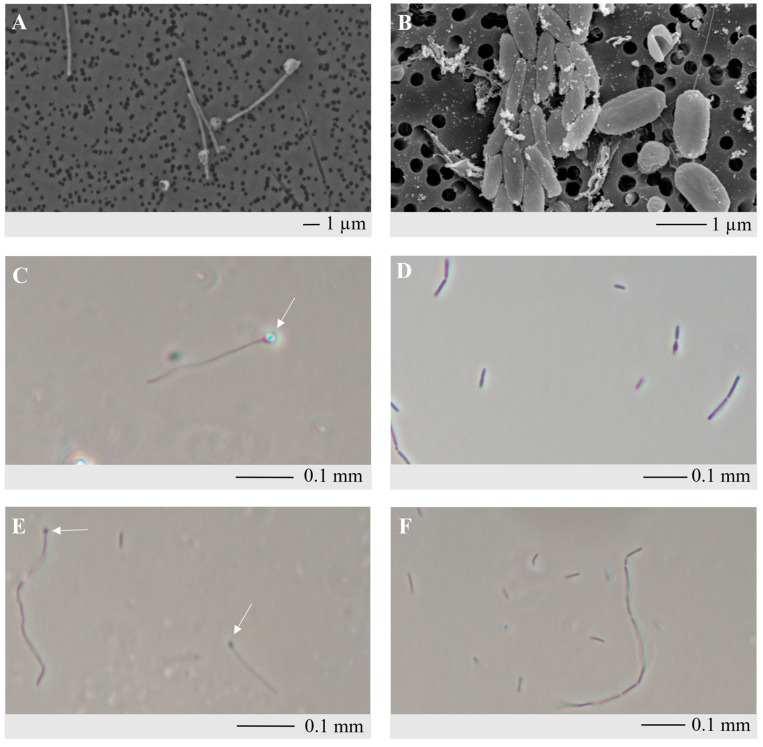
Scanning electron micrograph and phase-contrast microscopy of KAk grown at 70 °C. Scanning electron micrograph (**A**) and phase-contrast micrograph (**C**,**E**) of colonies grown on MMF with feathers include refractile (**C**) and non-refractile spores (**E**) that are indicated by arrows. Scanning electron micrograph (**B**) and phase-contrast micrograph (**D**,**F**) of colonies grown on MMF with glucose.

After conducting three dilution-to-extinction series in MMF supplemented with yeast extract, a pure culture was obtained and designated as the KAk strain. No growth was observed on solid medium. When growing on sugars (CM-cellulose, starch, sucrose, lactose, arabinose, galactose, sorbitol, mannose, and glucose), the cells of strain KAk were rods of varying size, 0.2–0.7 µm in diameter and 1–20 µm in length. Under optimal conditions in a glucose-containing medium, the cells were predominantly observed singly, in pairs, or less frequently in chains, and the strain did not form endospores. However, under conditions involving growth with merino wool, feathers, or under suboptimal conditions, the cells of strain KAk exhibited a significantly elongated structure and thinner morphology, with lengths ranging from 6 to 8 µm, and spores were observed (Figure 1).

### 3.2. Phylogenetic Identification

**Figure 2:**
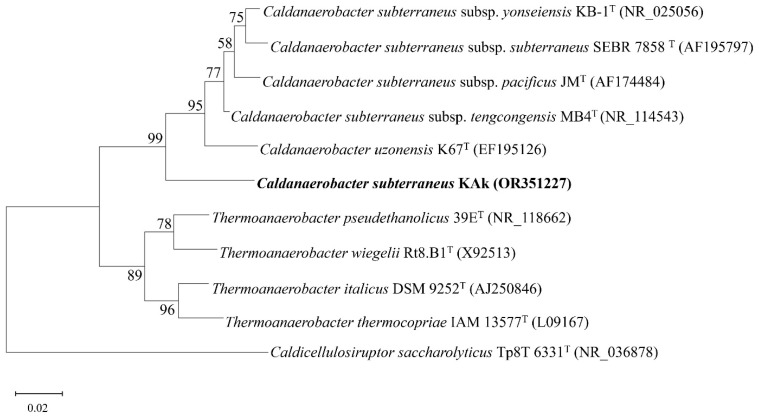
Phylogenetic maximum-likelihood tree based on 16S rRNA gene sequences of *Caldanaerobacter subterraneus* strain KAk (in bold) and representative members the family *Thermoanaerobacteraceae*. Accession numbers are shown in brackets. Bootstrap values are shown as percentages at the nodes. Bar, 0.02 changes per nucleotide position. *Caldicellulosiruptor saccharolyticus* Tp8T 6331^T^ (NR_036878) is used as the outgroup.

A maximum likelihood phylogenetic analysis of the 16S rRNA gene sequence (1470 bp) of strain Kak indicated placement within the genus *Caldanaerobacter*, with 98.84% sequence identity to the 16S rRNA gene sequence of *C*. *subterraneus* subsp. *tengcongensis* MB4^T^ in the family *Thermoanaerobacteraceae.* A phylogenetic tree based on the 16S rRNA gene sequence was generated for strain KAk and other representative members of the family *Thermoanaerobacteraceae*. The branching order indicated that *C*. *subterraneus* strain KAk formed a separate lineage, suggesting that strain KAk possibly represented a distinct subspecies (Figure 2).

### 3.3. Physiology

Yeast extract was not required for growth on carbohydrates, but it markedly accelerated the growth of strain KAk. Moreover, strain KAk demonstrated the ability to grow in the absence of almost all components of the MMF, except for yeast extract at a concentration of 0.01%. Turbidity due to cell growth was observed after incubating at 70 °C for one day with yeast extract. The KAk strain hydrolyzed sucrose, galactose, xylan, arabinose, xylose, glucose, peptone, CM-cellulose, and merino wool (Table 1). However, the growth was slower in the presence of starch and pyruvate than that of the other materials. Only *C. subterraneus* possessed peritrichous flagella, whereas strains KAk, *C. subterraneus* subsp. *yonseiensis*, and *Caldanaerobacter uzonensis* were capable of forming spores.

Strain KAk grew within a wide pH range, from 4.0 to 9.0. However, no growth was observed at NaCl concentrations exceeding 3%, with an optimal salt concentration of 0.4%. Furthermore, strain KAk grew within a temperature range of 55–80 °C, with an optimal temperature of 70 °C. According to API 20 A strips, the isolate demonstrated the ability to utilize 18 out of the 20 tested carbohydrates (Table S1). The enzymatic activity screening of strain KAk was positive for alkaline phosphatase, esterase (C4), esterase lipase (C8), lipase (C14), leucine arylamidase, valine arylamidase, cystine arylamidase, trypsin, α-chymotrypsin, acid phosphatase, naphthol-AS-BI-phosphohydrolase, α-glucosidase, β-glucosidase, and N-acetyl-β-glucosaminidase (Table S2). 

### 3.4. Feather Degradation

**Figure 3:**
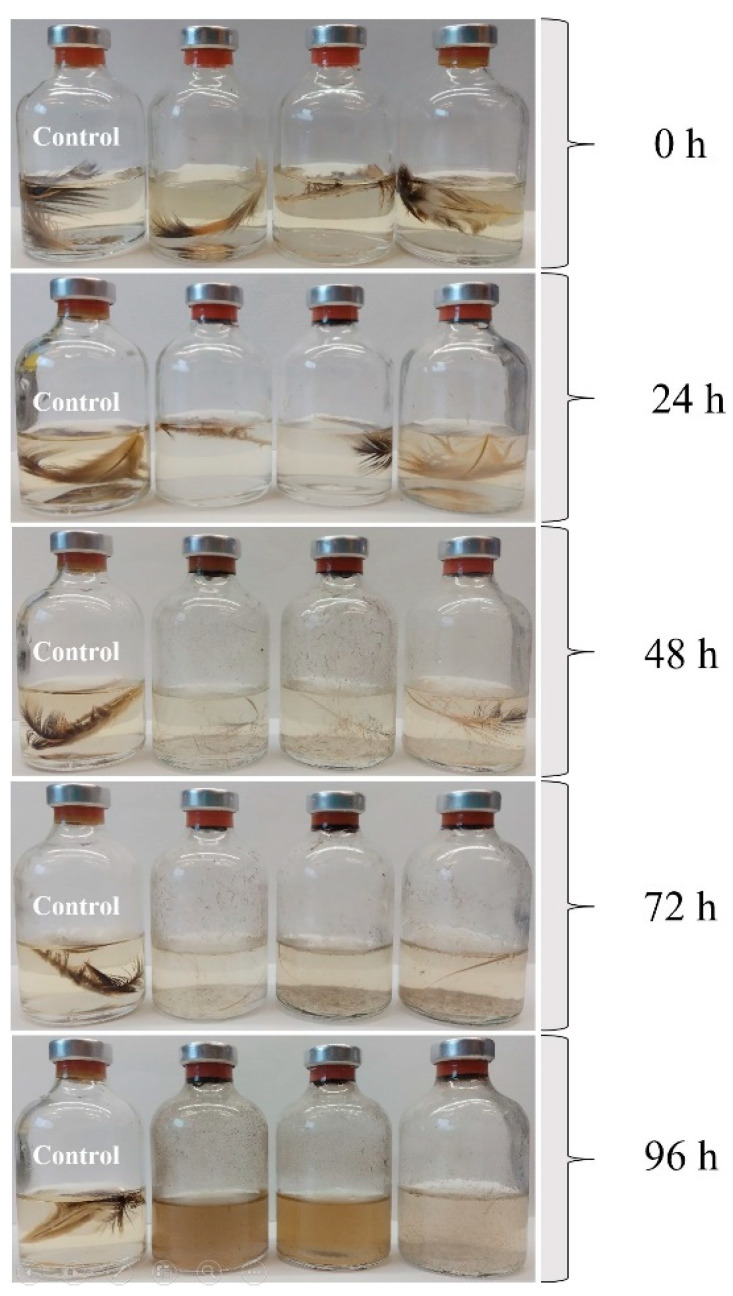
Degradation of native chicken feathers by the KAk strain at 70 °C. The control flask contains only medium with feathers but without inoculation. All flasks were maintained at the identical temperature throughout the incubation period.

The KAk strain exhibited the ability to degrade native chicken feathers in a medium containing solely chicken feathers as carbon and energy sources, achieving complete degradation within a period of 96 ± 2 h. This suggests that chicken feathers serve as the sole source of energy and carbon for the KAk strain. Optimal concentrations of yeast extract (0.1%) and NaCl (0.4%) were found to enhance keratinolytic activity, allowing the KAk strain to degrade feathers within three days. Remarkably, after four days, even the shaft, which is the more robust part of the feather, was completely degraded (Figure 3). However, both high (>1.7% yeast extract and >1% NaCl) and low concentrations (<0.05% yeast extract and <0.1% NaCl) inhibited feather degradation (Figure S1).

**Figure 4:**
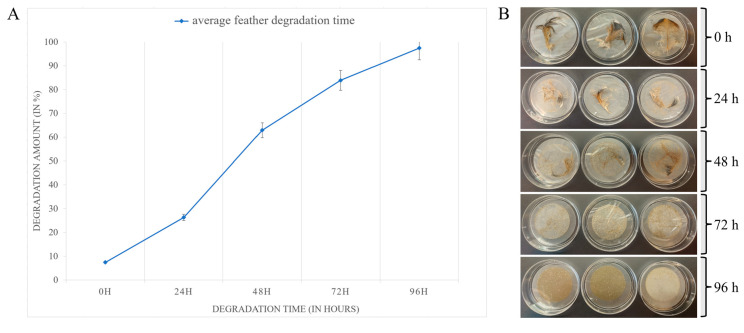
Feather degradation dynamics of feather keratin by *Caldanaerobacter* KAk. (**A**) Average feather degradation time and amount. (**B**) Prepared feathers for measuring the percentage of substrate weight loss after biodegradation by the KAk strain at different time points. The extent of feather biodegradation is quantified as the percentage weight loss relative to the initial dry weight of the substrate (before and after incubation).

After 24 h of fermentation, signs of degradation were evident in the intact chicken feathers (Figure 4B). After 48 h, approximately 60% ± 5% (Figure 4A) of feather degradation appeared as breakage of the barbule, revealing a softer internal fiber structure. By 72 h, the pinna rach and branches were almost completely degraded, with only the biggest part of the barb remaining, showing 84% ± 5% degradation (Figure 4A,B). Beyond 96 h, almost complete degradation, approximately 97% ± 5%, was achieved, with no observable structures remaining.

### 3.5. Genome Characteristics and Phylogenomics

The whole-genome shotgun sequencing project was deposited in GenBank under the accession number JAULJF000000000. The draft genome sequence was 2,425,203 bp distributed into 45 contigs, with an average GC content of 37.6%. The completeness of the KAk strain genome was 98.56%. Annotation with the NCBI Prokaryotic Genome Annotation Pipeline (PGAP) identified 2361 protein-coding sequences, with 242 sequences assigned to a subsystem based on annotation performed by the RAST server. Additionally, 52 tRNA genes were identified in the KAk genome (Table 2).

The majority of the annotated genes were associated with metabolic functions, including protein metabolism, amino acid anabolism, carbohydrate metabolism, DNA metabolism, and biosynthesis of cofactors or other secondary metabolites. Approximately 50 proteases were annotated in the KAk strain. Among these enzymes, cysteine, serine, and metalloproteases were the most prevalent. Furthermore, this approach led to the identification of genes predicted to be involved in resistance to environmental stressors, including osmotic stress, oxidative stress, periplasmic stress, and detoxification. Organisms appear to possess numerous defense mechanisms against toxic compounds and drugs in general. 

A pairwise phylogenomic comparison using the TYGS server revealed that the KAk strain was most closely affiliated with the *C. subterraneus* subsp. *younseiensis* type strain KB-1, with a dDDH identity value of 80.9 (Figure S2). *C. subterraneus* subsp. *subterraneus* DSM 13054^T^, *C. subterraneus* subsp. *tengcongensis* MB4^T^, and *C. subterraneus* subsp. *pacificus* DSM 12653^T^ also clustered with strains KAk and KB-1^T^, with dDDH values between 74.4 and 79.9%, exceeding the recommended species threshold value of 70%, thus confirming that the KAk strain belongs to *C. subterraneus*. However, the TYGS analysis indicated that strain KAk is sufficiently different from *C. subterraneus* subspecies to be placed in a separate subspecies cluster. This is further supported by specific metabolic differences. For instance, growth on carbohydrates did not necessitate yeast extract, and feathers could serve as the sole source of energy and carbon.

**Figure 5:**
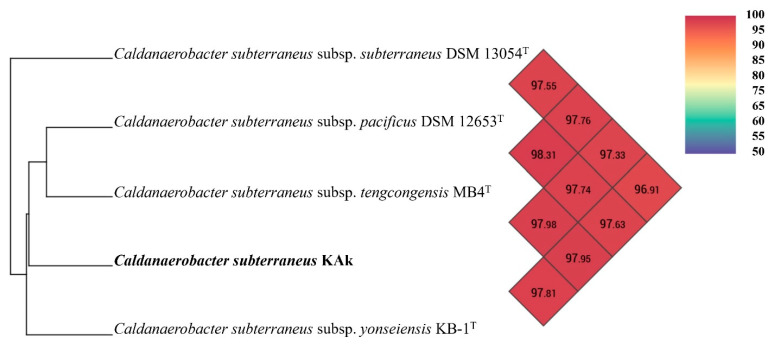
The heat map is generated using OrthoANI values calculated by the OAT software v0.93.1 between strain KAk and *Caldanaerobacter subterraneus* subspecies. Color codes represent the OrthoANI values in percentages.

An Ortho-ANI-based phylogenomic tree and heatmap corroborated the TYGS analysis, demonstrating clustering of the KAk strain with *C. subterraneus* subspecies (Figure 5), exhibiting ANI values ranging between 97.33 and 97.98%. 

**Figure 6:**
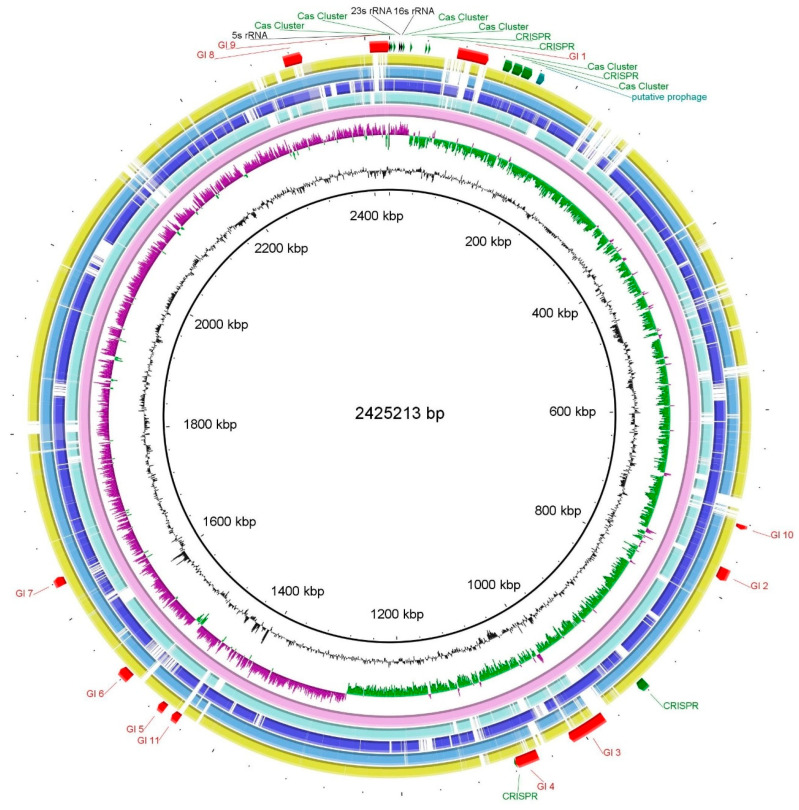
Circular representation of the genome of the KAk strain compared to those of C*aldanaerobacter subterraneus* subspecies. The rings were generated using the Blast Ring Image Generator (BRIG) cross-platform [35]. Before being utilized as the reference genome, the contigs of the KAk strain were arranged by Mauve [40], employing the complete genome sequence of *C. subterraneus* subsp. *tengcongensis* MB4^T^ as template. Rings from inside to outside: G+C content (black); GC skew (−, purple; +, green); *C. subterraneus* strain KAk (pink); *C. subterraneus* subsp. *subterraneus* DSM 13054^T^ (pale blue); *C. subterraneus* subsp. *pacificus* DSM 12653^T^ (blue); *C. subterraneus* subsp. *tengcongensis* MB4^T^ (turquoise); and *C. subterraneus* subsp. *yonseiensis* KB-1^T^ (citrine). The positions of genomic islands (indicated red arcs), rRNA operons (indicated black arcs), putative prophages (indicated teal), and CRISPR/Cas9 clusters (indicated green) are also marked. Genome sequence accession numbers: JAULJF000000000 (KAk); SLWU01000001 (DSM 13054^T^); ABXP00000000 (DSM 12653^T^); AE008691 (MB4^T^); and AXDC01000000 (KB-1^T^).

The Blast Ring Image Generator (BRIG) was employed for genome comparison between strains KAk and *C. subterraneus* (Figure 6). Prior to serving as a reference genome for BRIG, contigs from the KAk strain were rearranged and merged using Mauve [37] with the *C. subterraneus* subsp. *tengcongensis* MB4^T^ as the template. Furthermore, 11 putative genomic islands (GIs) were detected using the IslandViewer web server (www.pathogenomics.sfu.ca/islandviewer/; accessed on 28 May 2024) [38]. The sizes of the 11 putative GIs varied from 4935 bp (GI 10) to 33,022 bp (GI 1). GI 1, the largest island, contained 30 genes, 85.8% of which were hypothetical genes, whereas GI 10, the smallest island, contained only 5 genes. Compared to the average GC content of the KAk genome, all 11 GIs exhibited lower GC contents, ranging from 32.2% to 44.2%. A single prophage and 11 CRISPR/Cas elements were detected using the PHASTER pipeline [39] and CRISPRCasFinder [40], respectively (Figure 6).

## 4. Discussion

The new isolate characterized in this study was classified as *C. subterraneus*. However, minor distinctions from the *C. subterraneus* subspecies were observed, indicating that the KAk strain warrants classification as a distinct subspecies. The strain exhibited robust growth on a variety of substrates, including sucrose, galactose, xylan, arabinose, xylose, glucose, peptone, CM-cellulose, D-mannitol, D-lactose, D-maltose, salicin, gelatin, esculin ferric citrate, glycerol, D-cellobiose, D-mannose, D-melezitose, D-raffinose, D-sorbitol, L-rhamnose, and D-trehalose. Additionally, the KAk strain was able to degrade Merino wool and native chicken feathers. Furthermore, yeast extract was not required for carbohydrate growth. Notably, the KAk strain was capable of growing in media containing only yeast extract or chicken feathers, a unique characteristic compared to other members of the genus *Caldanaerobacter* [2,4,5,7].

KAk displays a temperature optimum of 70 °C and is capable of almost completely degrading chicken feathers after 96 h. Analysis of the genome of *C. subterraneus* KAk allowed identification of several potential keratinase enzyme candidates. Among *Caldanaerobacter* species, only the genomes of *C. subterraneus* (KB-1, MB4^T^, and DSM 12653^T^) have been reported to contain genes encoding proteases [8,41]. However, similar to other *Caldanaerobacter* members, *C. subterraneus* subsp. *younseiensis* KB-1^T^ was only able to hydrolyze type I and II collagen and only partially hydrolyzed type IV collagen, but not any type of keratin [42]. 

In addition to the physiological differences mentioned above, phylogenetic analyses of the KAk strain showed that it belonged to the same species group as *C. subterraneus* subsp. *younseiensis* KB-1^T^, *C. subterraneus* subsp. *subterraneus* DSM 13054^T^, *C. subterraneus* subsp. *tengcongensis* MB4^T^, and *C. subterraneus* subsp. *pacificus* DSM 12653^T^. Although the KAk strain belongs to *C. subterraneus*, both TYGS and OrthoANI analyses indicated notable differences between the four subspecies, suggesting that it may warrant classification as a novel and distinct subspecies.

## 5. Conclusions

The keratinolytic potential of the newly isolated *C. subterraneus* KAk was assessed, demonstrating efficient degradation of feather keratin within three days at temperatures ranging from 65 to 70 °C. Notably, the KAk strain exhibited a unique ability to thrive in media containing only yeast extract or chicken feathers, distinguishing it from other members of the genus *Caldanaerobacter.* This versatility underscores its adaptability and suggests its potential importance in diverse biotechnological contexts. Genomic analysis offered valuable insights into the feather keratin degradation capabilities of *C. subterraneus* KAk, highlighting its potential for industrial applications in poultry waste valorization for animal feed production and efficient waste management strategies for keratin-containing materials.

## 6. *Caldanaerobacter subterraneus* subsp. *keratinolyticus* subsp. nov.

*Caldanaerobacter subterraneus* subsp. *keratinolyticus* (ker.a.tino.lyti’cus N.L. masc. adj. *keratinolyticus,* referring to keratin, keratin-degrading bacteria)

The cells are anaerobic, spore forming, and non-motile. It can grow anaerobically at temperatures ranging from 55 to 80 °C, pH ranging from 4.0 to 9, and salinity ranging from 0 to 3% (w/v) NaCl. The optimal temperature and pH for its growth were approximately 70 °C and pH 6.8. No growth was observed on solid media. When growing on sugars (CM-cellulose, starch, sucrose, lactose, arabinose, galactose, sorbitol, mannose, and glucose), the cells of strain KAk were rods of varying size, 0.2–0.7 µm in diameter and 1–20 µm in length. Under optimal conditions in a glucose-containing medium, the cells were predominantly observed singly, in pairs, or less frequently in chains, and the strain did not form endospores. However, under conditions involving growth with merino wool, feather, or under suboptimal conditions, the cells of strain KAk exhibited a significantly elongated structure and thinner morphology, with lengths ranging from 6 to 8 µm, and spores were observed. The strain exhibited robust growth on a variety of substrates, including sucrose, galactose, xylan, arabinose, xylose, glucose, peptone, CM-cellulose, D-mannitol, D-lactose, D-maltose, salicin, gelatin, esculin ferric citrate, glycerol, D-cellobiose, D-mannose, D-melezitose, D-raffinose, D-sorbitol, L-rhamnose, and D-trehalose. Additionally, the KAk strain was able to degrade Merino wool and native chicken feathers within 3 days at temperatures ranging from 65 to 70 °C. Furthermore, yeast extract was not required for growth on carbohydrates. It produces alkaline phosphatase, esterase (C4), esterase lipase (C8), lipase (C14), leucine arylamidase, valine arylamidase, cystine arylamidase, trypsin, α-chymotrypsin, acid phosphatase, naphthol-AS-BI-phosphohydrolase, α-glucosidase, β-glucosidase, and N-acetyl-β-glucosaminidase. The draft genome sequence was 2,425,203 bp distributed into 45 contigs, with an average GC content of 37.6%. The genome sequences have been assigned the accession number JAULJF000000000 in GenBank/DDBJ/EMBL, and for the 16S rRNA gene, it is OR351227. The KAk was isolated from a hydrothermal spring near Zharkent City in the Almaty region of Kazakhstan.

## Figures and Tables

**Table 1:** Characteristics of strain KAk and all described *Caldanaerobacter* type strains.

Feature	1	2	3	4	5	6
Origin	Geothermal hot spring	Terrestrial hot spring	Oilfield water	Geothermal hot stream	Pacific Ocean hot vents	Terrestrial hot spring
Cell size (µm)	0.2–0.7 × 1–20	0.5–0.6 × 1–10	0.5–0.7 × 2–8	0.4–0.8 × 1–3	0.3 × 4–10	0.3–0.5 × 1.5–15
Spore	+	-	-	+	-	+
Motility	-	-	-	+	-	-
Flagella	-	-	Peritrichous	-	-	-
Temperature (°C)						
Range	55–80	50–80	40–75	50–85	50–80	50–75
Optimum	70	75	65	75	70	68–70
pH						
Range	4.0–9.0	5.5–9.0	6.0–8.5	4.5–9.0	5.8–7.6	4.8–8.0
Optimum	6.8	7.0–7.5	7.5	6.5	6.8–7.1	6.8
NaCl (%)						
Range	0–3	0–2.5	0–3	0–4	ND	0–2
Optimum	0.4	0.2	0	0	2–2.5	0.5
Starch hydrolysis	slow	+	+	+	+	+
Xylan degradation	+	-	+	-	ND	ND
Utilization of:						
Glucose	+	+	+	+	+	+
Sucrose	+	-	-	+	-	+
Xylose	+	-	+	+	ND	+
Arabinose	+	-	-	-	ND	+
Pyruvate	slow	-	+	ND	+	+
Galactose	+	+	+	+	+	+
Peptone	+	+	+	+	+	+
DNA G+C content (mol%)	37.6	33	41	36.6	33 ± 1	34.2 ± 0.5

1, Strain KAk; 2, *C. subterraneus* subsp*. tengcongensis* MB4^T^ [6]; 3, *C. subterraneus* subsp. *subterraneus* DSM 13054^T^ [3,4]; 4, *C. subterraneus* subsp. *yonseiensis* KB-1^T^ [7,8]; 5, *C. subterraneus* subsp. *pacificus* JM^T^ [5]; and 6, *C. uzonensis* K67^T^ [2]. ND, not determined; +, growth after 24 h incubation; -, no growth after 24 h incubation; and slow, growth after 48 h incubation.

**Table 2:** Genome statistics of *Caldanaerobacter subterraneus* genomes *.

Scientific Name	*C. subterraneus* KAk	*C. subterraneus* subsp. *subterraneus* DSM 13054^T^	*C. subterraneus* subsp. *pacificus* JM^T^	*C. subterraneus* subsp*. tengcongensis* MB4^T^	*C. subterraneus* subsp. *younseiensis* KB-1^T^
Genome size (Mb)	2.4	2.6	2.4	2.7	2.7
G+C content (%)	37.6	37.5	37.5	37.5	37.5
N50 (kb)	108.9	82.6	56.3	2.7	62.5
L50	8	11	13	1	14
Number of contigs	56	83	135	1	102
Number of coding sequences	2361	2613	2511	2588	2711
Number of tRNAs	52	56	49	55	59
5S rRNA	3	3	4	4	4
16S rRNA	2	5	4	4	5
23S rRNA	3	5	3	4	9
NCBI Accession no.	JAULJF000000000	SLWU00000000	ABXP00000000	AE008691	AXDC01000000

* Data are retrieved from GenBank [36].

## Data Availability

The complete genome sequence is available under GenBank accession number JAULJF000000000.

## Supplementary Materials

The following supporting information can be downloaded at: https://www.mdpi.com/article/10.3390/microorganisms12071277/s1, Figure S1: Effect of yeast extract concentration (A) and NaCl concentration (B) on keratinase activity of strain Caldanaerobacter KAk; Figure S2: Phylogenomic tree of C. subterraneus strain KAk and C. subterraneus subspecies and representative members of the genus Thermoanaerobacter.; Table S1: API 20 A fermentation patterns of isolate KAk. References [43,44,45] are cited in the supplementary materials.
