# Supplementary material for: Caldanaerobacter subterraneus subsp. keratinolyticus subsp. nov., a Novel Feather-Degrading Anaerobic Thermophile
Source: Microorganisms. 2024 Jun 23;12(7):1277. doi: 10.3390/microorganisms12071277 (PMC11278675; doi:10.3390/microorganisms12071277)
Supplement: Supplementary file 1 [file microorganisms-12-01277-s001.zip › microorganisms-3069367-supplementary.pdf]

SUPPLEMENTARY MATERIAL

Caldanaerobacter subterraneus subsp. *keratinolyticus* subsp. nov., a Novel Feather-Degrading Anaerobic Thermophile

Akzhigit Mashzhan, Aida Kistaubayeva, Rubén Javier-López, Akerke Bissenbay, Nils-Kåre Birkeland

Table S1. API 20 A fermentation patterns of isolate KAk.

Active ingredients	
L-tryptophane	-
urea	-
D-glucose	+
D-mannitol	+
D-lactose (bovine origin)	+
D-sucrose	+
D-maltose	+
salicin	+
D-xylose	+
L-arabinose	+
gelatin (bovine origin)	+
esculin ferric citrate	+
glycerol	+
D-cellobiose	+
D-mannose	+
D-melezitose	+
D-raffinose	+
D-sorbitol	+
L-rhamnose	+
D-trehalose	+

Table S2. API ZYM fermentation patterns of isolate KAk.

Active ingredients	
Alkaline phosphatase	+
Esterase (C 4)	+
Esterase Lipase (C 8)	+
Lipase (C 14)	+
Leucine arylamidase	+
Valine arylamidase	+
Cystine arylamidase	+
Trypsin	+
α-chymotrypsin	+
Acid phosphatase	+
Naphthol-AS-BI-phosphohydrolase	+
α-galactosidase	-
β-galactosidase	-
β-glucuronidase	-
α-glucosidase	+
β-glucosidase	+
N-acetyl-β-glucosaminidase	+

α-mannosidase	-
α-fucosidase	-

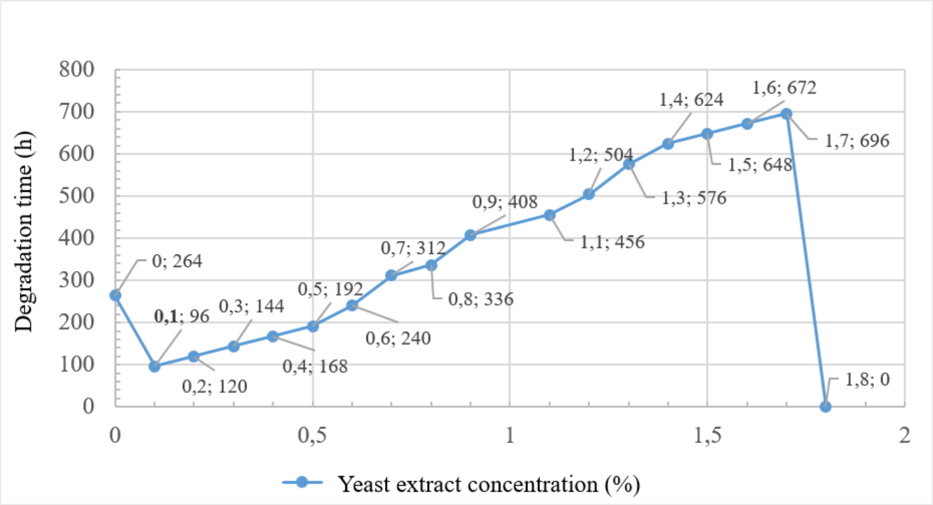

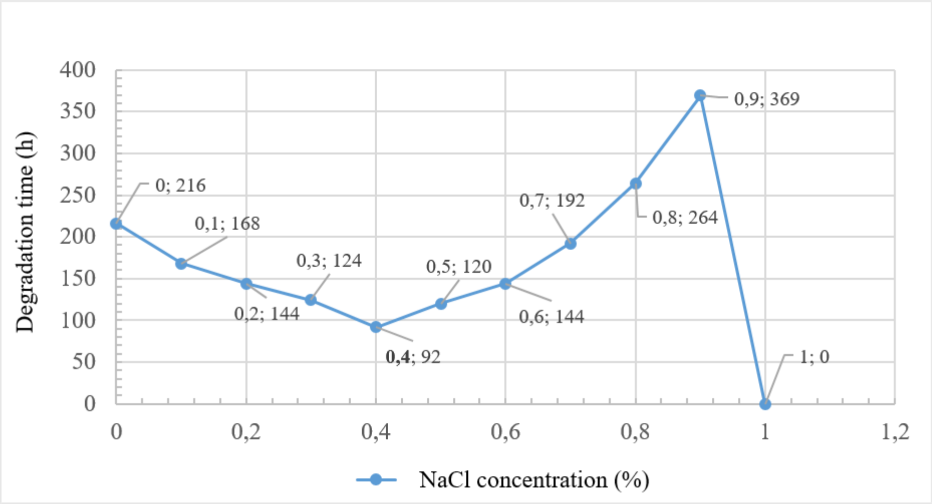

Figure S1. Effect of yeast extract concentration (A) and NaCl concentration (B) on keratinase activity of strain Caldanaerobacter KAk

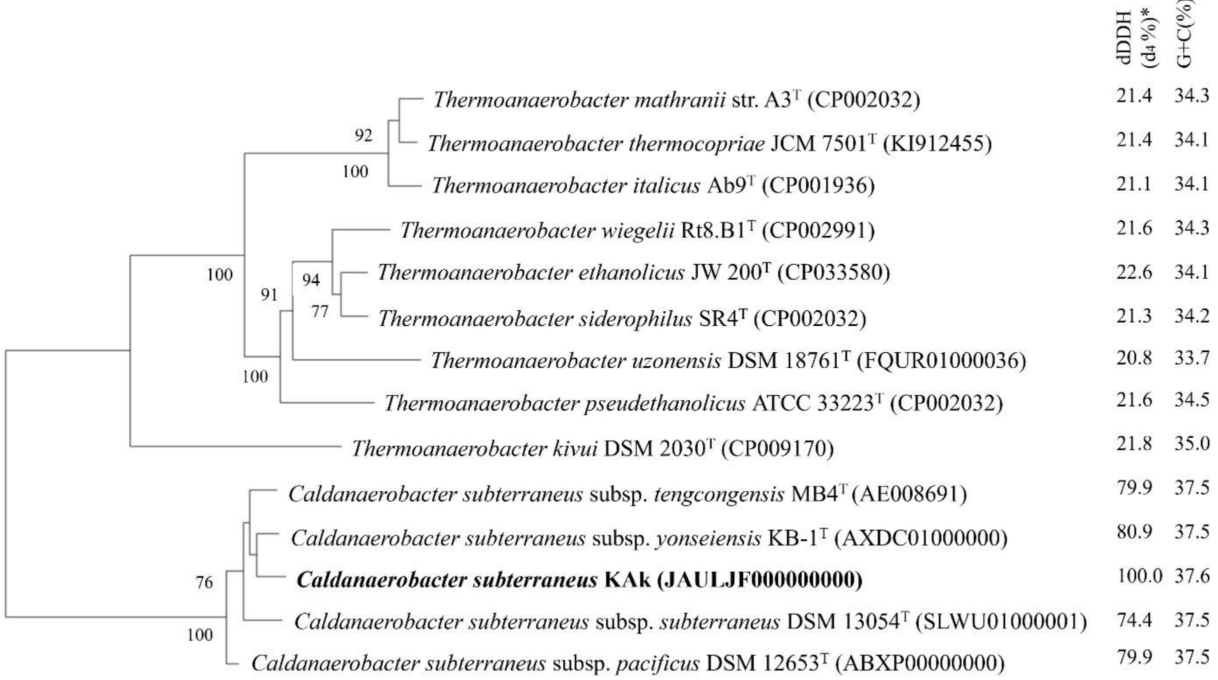

Figure S2. Phylogenomic tree of *C. subterraneus* strain KAk and *C. subterraneus* subspecies and representative members of the genus *Thermoanaerobacter*. The tree was inferred using FastME 2.1.6.1 [1] from the Genome Blast Distance Phylogeny (GBDP). The branch lengths are scaled according to the GBDP distance formula d5. The numbers above branches represent GBDP pseudo-bootstrap support values greater than 60% from 100 replications, with an average branch support of 95.9%. The tree was rooted at the midpoint [2] and visualized with PhyD3 [3]. *The dDDH values (formula 2) and G+C contents are displayed on the right.

References

1. Lefort, V.; Desper, R.; Gascuel, O. FastME 2.0: A Comprehensive, Accurate, and Fast Distance-Based Phylogeny Inference Program. *Mol. Biol. Evol.* **2015**, 32(10), 2798-2800.
2. Farris, J.S. Estimating Phylogenetic Trees from Distance Matrices. Am. Nat. **1972**, 106, 645-668.
3. Kreft, L.; Botzki, A.; Coppens, F.; Vandepoele, K.; Van Bel, M. PhyD3: a phylogenetic tree viewer with extended phyloXML support for functional genomics data visualization. *Bioinformatics*. **2017**, 33(18), 2946-2947.
